# Supplementary material for: Tuning of the Hanle effect from EIT to EIA using spatially separated probe and control beams
Source: Sci Rep. 2018 May 14;8:7525. doi: 10.1038/s41598-018-25832-8 (PMC5951910; doi:10.1038/s41598-018-25832-8)
Supplement: Supplementary file 1 — Supplementary Information [file 41598_2018_25832_MOESM1_ESM.pdf]

Tuning of the Hanle effect from EIT to EIA using spatially separated probe and control beams

Mangesh Bhattarai[1], Vineet Bharti[1], and Vasant Natarajan[1,*]

[1]Department of Physics, Indian Institute of Science, Bangalore-560012, India
[*]vasant@physics.iisc.ernet.in

Supplementary information

Here, we discuss the method to approximate the Rabi frequencies for theoretical analysis. The saturation intensity (I_s) is not the same for atoms moving with different longitudinal velocities.

The population of excited state after the interaction of atoms with light is given as:

$$\rho_{22} = \frac{\Omega^2/4}{\delta^2 + \Omega^2/2 + \Gamma^2/4} \tag{1}$$

where δ is the detuning of light from resonance, Ω is the Rabi frequency, and Γ is the natural linewidth of excited state. For light field on resonance ($\delta = 0$) and atoms moving with v, Eq. (1) can be written as:

$$\rho_{22}(v) = \frac{\Omega^2/4}{(kv)^2 + \Omega^2/2 + \Gamma^2/4} \tag{2}$$

The excited state population by considering Maxwell-Boltzmann (MB) velocity distribution of atoms is

$$\rho_{22} = \int_{-\infty}^{+\infty} \rho_{22}(v) f(v) dv, \tag{3}$$

where

$$f(v) = \sqrt{\frac{m}{2\pi k_B T}} \exp\left(\frac{-mv^2}{2k_B T}\right) \tag{4}$$

We solve for the Ω/Γ at saturation by equating Eq. (3) to 1/4. In our theoretical model, we have ignored the longitudinal velocity distribution. Therefore, the

effective Rabi frequency for the model assuming atoms with zero longitudinal velocities—calculated by assuming the ratio I/I_s is same for both model and experiment—is given as

$$(\Omega/\Gamma)_{\mathrm{model}} = \frac{1}{\sqrt{2}} \frac{(\Omega/\Gamma)_{\mathrm{expt}}}{(\Omega/\Gamma)_{I_s,\mathrm{MB}}} \tag{5}$$
